# Cooperative Detection-Oriented Formation Design and Optimization of USV Swarms via an Improved Genetic Algorithm

**DOI:** 10.3390/s25103179

**Published:** 2025-05-18

**Authors:** Rui Liang, Dingzhao Li, Haixin Sun, Liangpo Hong

**Affiliations:** 1 School of General Education, Xiamen City University, Xiamen 361005, China; liangrei@163.com; 2 School of Information, Xiamen University, Xiang’an District, Xiamen 361102, China; lidingzhao@stu.xmu.edu.cn (D.L.); 15695902050@163.com (L.H.); 3 Key Laboratory of Southeast Coast Marine Information Intelligent Perception and Application, Ministry of Natural Resources, Zhangzhou 363000, China

**Keywords:** unmanned surface vehicle (USV), formation optimization, genetic algorithm, multi-objective optimization, marine environment

## Abstract

Efficient and adaptive formation planning is critical for unmanned surface vehicle (USV) swarms equipped with sensor networks and smart sensors to perform cooperative detection tasks in complex marine environments. Existing formation optimization methods often overlook the nonlinear coupling between sensor-based detection performance, communication constraints, and obstacle avoidance. We propose a multi-objective formation optimization framework based on an improved genetic algorithm that simultaneously considers the detection coverage area, forward detection width, inter-agent communication, and static obstacle avoidance. We formulate a probabilistic cooperative detection model, introduce normalized detection efficiency indicators, and embed multiple geometric and environmental constraints into the optimization process. Simulation results show that the proposed method significantly improves the spatial efficiency of cooperative sensing, yielding a 32.76% increase in effective coverage area and 20.97% improvement in forward detection width compared to unoptimized formations. This strategy, supported by multi-sensor positioning and navigation, offers a robust and generalizable approach for intelligent maritime USV deployment in dynamic, multi-constraint scenarios.

## 1. Introduction

Unmanned surface vehicles (USVs) are a cornerstone of modern maritime technology, demonstrating significant potential in applications such as environmental monitoring, oceanographic surveying, military reconnaissance, and commercial shipping [1]. These autonomous platforms operate without direct human intervention, leveraging smart sensors and sensor networks integrated with advanced signal processing to navigate and perform tasks in complex marine environments [2]. With the growing demand for USV applications, particularly in scenarios requiring multi-vehicle collaboration, sensor technology has become critical for enabling formation-based detection. By utilizing AI-enabled sensors and multi-sensor positioning and navigation, USVs achieve enhanced cooperative sensing and robust performance in dynamic maritime settings, making them indispensable for environmental sensing applications.

USV formation detection entails multiple USVs operating collaboratively in specific configurations to achieve greater task efficiency than single-vehicle operations. Formation arrangements are critical for tasks such as collaborative surveillance, search and rescue, and synchronized data acquisition, as they enable broader area coverage, provide observational redundancy, and enhance mission robustness [3]. For example, in marine pollution monitoring, USV formations can utilize distributed sensor networks to enable real-time data sharing, thereby improving monitoring efficiency [4]. However, the design and optimization of USV formations face numerous challenges. First, collision avoidance among USVs and with static or dynamic obstacles requires precise path planning and real-time adjustments [5]. Second, optimizing energy consumption is essential, as USVs typically demand sustained high-efficiency operation during extended missions [6]. Furthermore, formations must adapt to uncertainties in the marine environment, such as currents, waves, and communication disruptions, which place stringent demands on algorithm robustness and scalability [7].

To address these challenges, researchers have developed various algorithms and methodologies. For instance, Ant Colony Optimization (ACO), combined with fuzzy logic and clustering techniques, has been employed to optimize multi-objective path planning, balancing path length, energy consumption, and obstacle avoidance requirements [4]. Biologically Inspired Neural Networks (BINNs) have also been applied to coverage path planning, demonstrating high efficiency in complex marine environments [8]. Additionally, formation control strategies based on virtual structures and artificial potential fields enable USVs to maintain formation by tracking a virtual leader while using potential field methods for obstacle avoidance [9]. Burlutskiy et al. [10] established a leader–follower formation structure for Autonomous Underwater Vehicles (AUVs), optimizing formation configurations from the perspective of dynamic efficiency to achieve a balanced optimal formation that minimizes energy consumption, maximizes coverage, and reduces communication power. Liang et al. [11] analyzed the drag sources in AUV formation navigation based on propeller structures, providing an optimal formation configuration for minimal drag. He et al. [12] conducted numerical modeling to compare drag and energy consumption across different formation configurations, offering insights into energy-efficient designs. Similarly, Dong et al. [13] proposed an energy-saving formation strategy for multiple USVs navigating on the water surface, addressing hydrodynamic energy consumption in longitudinal and lateral configurations and optimizing formations based on spacing intervals. Zou et al. [14] focused on maximizing underwater target search efficiency, proposing a particle swarm optimization algorithm to optimize the formation of heterogeneous USV and Unmanned Underwater Vehicle (UUV) fleets.

In the context of USV formation-based detection, neglecting detection probability modeling introduces significant mission risks, such as coverage gaps and sensing failures. Coverage gaps arise when certain regions of interest are not adequately covered due to improper formation planning. Sensing failures occur when the positioning of USVs results in a failure to detect objects within the expected range, which could lead to mission inefficiencies or even failures in critical tasks like search and rescue operations. These limitations underscore the importance of incorporating accurate detection probability models in USV formation planning to ensure robust performance in dynamic and challenging maritime environments [1,9,14].

In recent years, the research on USV formation planning has evolved significantly across three key themes: energy-aware control, obstacle-aware formation, and sensing-oriented planning. 1. Energy-Aware Control: Energy-efficient formation planning has been a critical focus, with methods aiming to minimize energy consumption while maximizing mission success. Liang et al. (2020) [11] analyzed the drag sources in AUV formation navigation to provide an optimal formation configuration for minimal energy loss, and Zou et al. (2021) [14] proposed strategies for USV and UUV formation optimization with energy-saving strategies in dynamic conditions. 2. Obstacle-Aware Formation: A significant challenge in USV formation planning is the ability to avoid obstacles in dynamic environments. Niu et al. (2016) [5] developed algorithms for collision avoidance, while Zhu et al. (2024) [3] explored advanced techniques for USVs to navigate in challenging maritime environments, which is essential for maintaining the safety and effectiveness of multi-vehicle operations. 3. Sensing-Oriented Planning: The integration of probabilistic sensing models has proven vital in improving detection performance. Tang et al. (2023) [8] proposed a probabilistic model to enhance coverage for marine pollution monitoring, while Ntakolia et al. (2023) [4] developed a path planning algorithm that incorporates sensing capabilities for more efficient USV formation-based detection.

As summarized above, although various USV formation patterns have been proposed through computer simulations and experimental validations, there is still a lack of effective design methodologies to ensure the rationality and optimality of such formations. Preliminary studies on USV formation planning have been conducted; however, most existing research primarily focuses on energy consumption during formation navigation. Relatively few works consider the formation configuration from the perspective of improving cooperative operational efficiency among USVs. To this end, this study focuses on the optimal formation planning of USV formations for marine detection missions. Specifically, formation design and optimization strategies for USV fleets are investigated. To maximize the overall detection performance of USV formations under obstacle-constrained environments, a formation design and optimization strategy based on an improved genetic algorithm is proposed. This approach aims to obtain the optimal formation configuration of the USV fleet under multiple constraints. The main contributions of this study are as follows:

- For the formation planning problem in USV swarm detection tasks, we introduce a novel multi-constraint formation optimization mathematical model aimed at maximizing comprehensive detection efficiency. By enhancing the genetic algorithm with elite selection and large mutation strategies, the proposed framework significantly improves the global search capability and resistance to local optima, providing an efficient solution for USV formation design in complex marine environments.
- By incorporating elite selection and large mutation strategies into genetic operators, we improve the convergence performance and solution quality of the traditional genetic algorithm. This approach effectively mitigates the risk of local optima, ensuring high-quality formation solutions under multiple constraints (e.g., detection coverage, communication limits, and obstacle avoidance), thereby supporting the robustness and adaptability of USV formations.
- Through experimental validation, we demonstrate that the improved genetic algorithm successfully derives optimal USV formation configurations, significantly enhancing comprehensive detection efficiency. The results highlight superior resistance to local optima, achieving improved detection coverage and efficiency compared to unoptimized formations, offering a generalizable strategy for intelligent maritime USV deployment.

The remainder of this paper is organized as follows: Section 1 constructs the USV detection model, focusing on sensor-based cooperative sensing. Section 2 formulates the mathematical model for formation optimization, integrating sensor network constraints. Section 3 presents the formation optimization strategy based on an improved genetic algorithm. Section 4 discusses the experimental results. Section 5 provides the conclusions.

## 2. USV Detection Model Construction

In order to rapidly and accurately evaluate the sensing capability of USV formations, it is necessary to construct a model to estimate detection effectiveness within the regional coverage area. For multi-agent platforms engaged in cooperative detection, many previous studies have addressed the establishment of detection boundary models and perception fields.

This section adopts a discretized gridded representation of the maritime environment. A grid-based map is constructed over the sea area, where each grid cell’s center represents a detection point for the USV formation. Based on the acoustic detection model, detection probabilities are computed as a function of distance to the target, and used as the evaluation basis for cooperative detection performance of the formation.

Table 1 provides a summary of the acoustic model parameters, including source level (SL), transmission loss (TL), noise level (NL), directivity index (DI), sound quality factor (FOQ), variance of Gaussian noise ($sigma^{2}$), and false alarm probability ($P_{f}$). We agree that providing a more detailed explanation and citing appropriate references for these parameters would enhance both the clarity and physical understanding of the model.

### 2.1. Gridding Method

The gridding method was proposed by researchers such as Elfes and Moravec [15]. It is a widely used technique for modeling spatial environments in robotics. The core idea is to divide a given region into equally sized square cells, with each cell representing an area of uniform resolution and coverage. Grid cells act as atomic units for environmental modeling and information updating.

The method mainly consists of two steps. First, the region of interest is uniformly divided according to the specified resolution, typically based on coordinates or distance steps. Second, each grid cell is assigned an attribute value. For detection modeling, this attribute may represent the detection probability derived from the target-to-sensor distance, and is associated with the USV position as the origin.

Thus, the detection probability field can be efficiently represented using a matrix of grid cell values, enabling real-time analysis of coverage, redundancy, and blind zones in USV cooperative sensing tasks.

### 2.2. Cooperative Detection Model for USV Formations

To construct a cooperative detection model for the USV formation, the detection process of a single USV must be established first. Given the energy constraints and mobility limitations of USVs in practical missions, passive acoustic sensing is commonly adopted. The received signal-to-noise ratio (SNR) can be expressed as

(1) SNR=SL−TL−(NL−DI)

where SL denotes the source level, TL is the transmission loss, NL represents the noise level, and DI is the directivity index. In underwater acoustics, TL typically follows a logarithmic model:

(2) $$TL=20\log_{10}\left(r\right)$$

where *r* denotes the distance (in meters) between the USV and the target.

Substituting Equation (2) into Equation (1), and using the constant sound quality factor FOQ=SL−(NL−DI), we obtain

(3) $$\mathrm{SNR}=FOQ-20\log_{10}\left(r\right)$$

To model the probability of target detection, the detection threshold is compared with the SNR in the context of a Gaussian signal distribution. The detection probability function is defined as

(4) $$p\left(x\right)=\left(\frac{x}{\sigma^{2}}\exp\left(-\frac{SNR+x}{2\sigma^{2}}\right)\right)\cdot I_{0}\left(\frac{\sqrt{2\cdot SNR\cdot x}}{\sigma }\right),\;x>0,$$

where *x* denotes the observed energy, $\sigma^{2}$ is the variance of Gaussian noise, and $I_{0}\left(\cdot \right)$ is the modified Bessel function of the first kind. The detection probability is then the integral of p(x) above a detection threshold DT:

(5) $$P_{d}=\int_{DT}^{\infty }p\left(x\right)\;dx=Q\left(\sqrt{2\cdot SNR},\sqrt{2\ln\frac{1}{P_{f}}}\right)$$

where Q(a,b) is the Marcum-Q function, and $P_{f}$ is the false alarm probability.

The detection threshold DT can be approximated as

(6) $$DT\approx 10\log_{10}\left(\log_{e}\frac{1}{P_{f}}\right)-0.8$$

**Figure 1:**
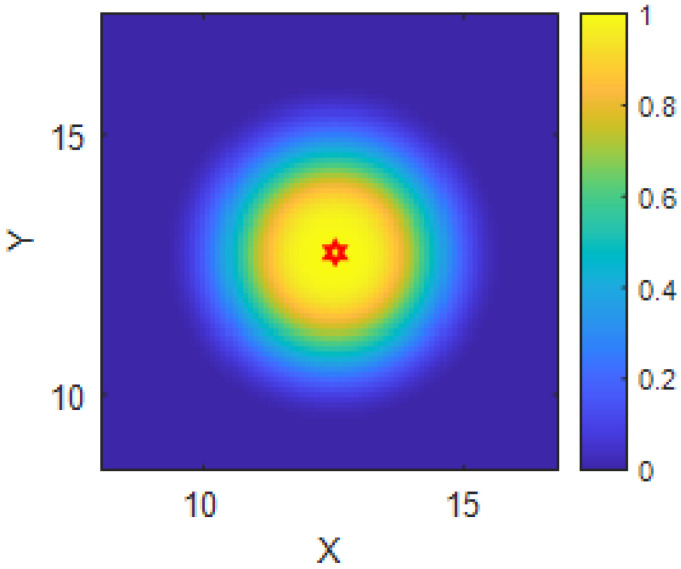
Single USV target detection probability distribution (Red star: single USV).

According to Equations (5) and (6), the detection probability of a single USV is determined jointly by SNR and the detection threshold. This probabilistic model enables the estimation of cooperative detection capability when multiple USVs are deployed in formation by aggregating detection probabilities across grid cells in the environment, as shown in Figure 1.

When multiple USVs are deployed in formation, the target detection probability at a given location is not simply the arithmetic sum of the individual USV detection probabilities. Instead, a probabilistic fusion model is used to characterize the joint sensing effect. The cumulative detection probability at point *r* is expressed as

(7) $$P\left(r\right)=1-\prod_{i=1}^{N}\left[1-P_{i}\left(r\right)\right]$$

where P(r) is the cooperative detection probability at position *r*, *N* is the total number of USVs, and $P_{i}\left(r\right)$ represents the individual detection probability of the *i*-th USV at position *r*.

**Figure 2:**
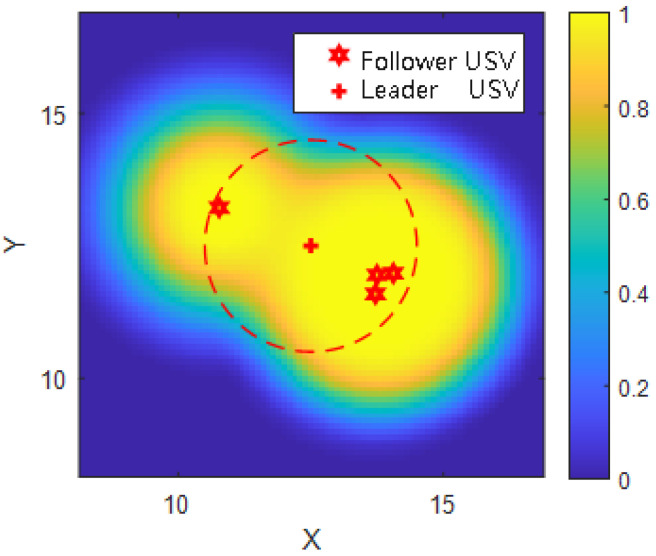
Cooperative detection probability distribution of a USV formation, the red dotted circle indicates the effective communication range of the leader USV.

As shown in Figure 2, the USV formation achieves spatial detection coverage through probabilistic fusion, where regions of overlapping influence contribute higher cumulative detection probability.

## 3. Formation Optimization Mathematical Model

Different formation patterns are composed of *N* follower USVs located at different positions. By solving the position of each follower relative to the leader, any desired formation configuration can be represented. In the optimization framework, the follower USVs’ positions are treated as decision variables, while the leader USV serves as the formation reference center.

Let the formation state vector be defined as

(8) $$X\left(r,\theta \right)={\left(r_{1},r_{2},\ldots ,r_{N},\theta_{1},\theta_{2},\ldots ,\theta_{N}\right)}^{T}$$

Each polar coordinate $\left(r_{i},\theta_{i}\right)$ is converted into Cartesian coordinates according to

(9) $$\left\{\begin{array}{c} r_{i}=\sqrt{{\left(x_{i}-x_{c}\right)}^{2}+{\left(y_{i}-y_{c}\right)}^{2}}\\ \theta_{i}=\arctan\left(\frac{y_{i}-y_{c}}{x_{i}-x_{c}}\right) \end{array}\right.,\;i=1,2,\ldots ,N$$

where *N* is the number of follower USVs, $\left(x_{c},y_{c}\right)$ is the coordinate of the leader USV (serving as the communication reference center), and $\left(x_{i},y_{i}\right)$ denotes the position of the *i*-th follower USV in global coordinates.

To ensure effective communication and control, it is necessary to constrain the positions of follower USVs to remain within a limited range. This is usually expressed as

(10) $$R_{\mathrm{safe}}\leq r_{i}\leq R_{\max},\;\theta_{i}\in \left[0,2\pi \right]$$

where $R_{\mathrm{safe}}$ is the minimum safety communication distance, and $R_{\max}$ is the maximum allowable radius from the leader to the follower USVs. These constraints ensure the feasibility of the formation design in terms of both connectivity and safety margins.

### 3.1. Fitness Function

As introduced in the previous section, the cooperative detection model for a USV formation aims to capture the probabilistic relationship between sensing coverage and spatial target distribution. In practical settings, when the detection probability P(r,θ) at a given location is below a predefined threshold $P_{t}$, the point is considered undetectable. Therefore, the binary detection value *n* at a given point is defined as

(11) $$n=\left\{\begin{array}{cc} 1, & P\left(r,\theta \right)\geq P_{t}\\ 0, & P\left(r,\theta \right)<P_{t} \end{array}\right.$$

To evaluate the directional detection capability, the effective forward sensing width of the formation is also considered a key indicator. Assuming the leader USV is positioned at the coordinate origin, and the Y-axis represents the forward direction of motion, the forward effective width is defined as

(12) $$L_{\mathrm{wid}}=\max\left\{\sum_{x_{i}}n_{i}^{t}\;|\;y_{i}\in Y^{t}\right\}\cdot \mu$$

Here, $x_{i}$ denotes the set of x-coordinates in the detection region, $Y^{t}$ is the subset of y-coordinates at scan row *t*, $n_{i}^{t}$ is the binary detection indicator from Equation (11), and μ is the grid step size (resolution).

The width calculation proceeds as follows:

- First, identify the detection region $A_{s}$ above the threshold $P_{t}$ based on the cooperative probability distribution.
- Then, project all grid cells in $A_{s}$ onto the Y-axis, and compute the maximum lateral (X-direction) length, where n=1.
- Finally, multiply the maximum grid count by resolution μ to obtain $L_{\mathrm{wid}}$.

**Figure 3:**
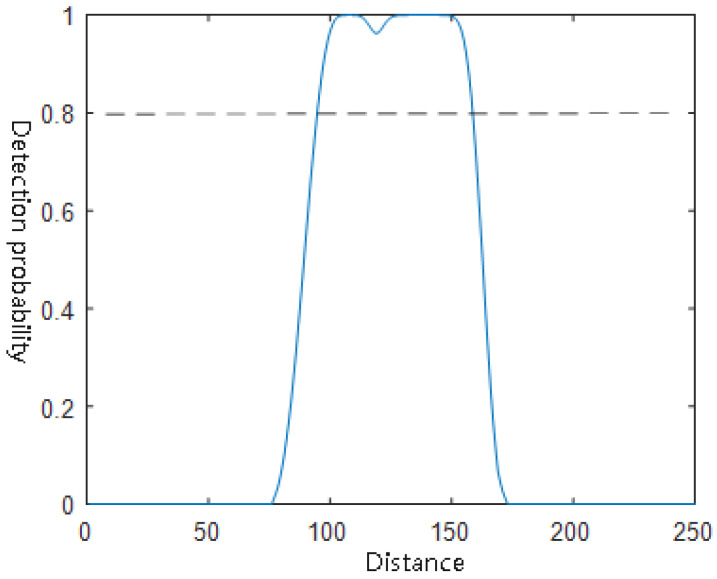
Forward detection probability width curve for multiple USVs.

As observed in Figure 2 and Figure 3, the left peak in the frontal detection probability curve is primarily contributed by a single USV, while the right peak results from the cooperative detection of three closely spaced USVs. Consequently, the right peak exhibits a broader distribution than the left peak. For example, assuming a detection threshold $P_{t}=0.8$, and grid resolution μ=0.1 km, if there are six adjacent detectable cells in a scan line, the computed effective detection width is

(13) $$L_{\mathrm{wid}}=6\times 0.1=0.6\;\mathrm{km}$$

This metric enables the quantification of how “wide” the sensing footprint is in the frontal direction, providing an intuitive geometric assessment of the USV formation’s sensing configuration.

To quantify the spatial performance of USV cooperative sensing, we define the effective detection area $S_{\mathrm{cov}}$ as

(14) $$S_{\mathrm{cov}}=\sum_{i\in X,j\in Y}n_{ij}\cdot \mu^{2}$$

To normalize detection indicators and facilitate optimization, a scaled version of $L_{\mathrm{wid}}$ is defined as

(15) $$F_{1}=\frac{10(L_{\mathrm{wid}}-2r_{t})}{2\left(R+r_{t}\right)-2r_{t}}$$

and the scaled version of effective coverage is defined as

(16) $$F_{2}=\frac{10(S_{\mathrm{cov}}-\pi r_{t}^{2})}{\pi {\left(R+r_{t}\right)}^{2}-\pi r_{t}^{2}}$$

The combined weighted objective function is then expressed as

(17) $$F=u_{1}\cdot F_{1}+u_{2}\cdot F_{2}$$

where $u_{1}+u_{2}=1$, and $u_{1},u_{2}$ are the priority weights assigned to the width and area optimization, respectively.

The optimization objective becomes

(18) $$\max F\left(X\left(r,\theta \right)\right)=\max\left(u_{1}\cdot F_{1}+u_{2}\cdot F_{2}\right)$$

### 3.2. Multiple Constraints

Beyond the aforementioned detection-based objectives, follower USV positions must also satisfy multiple physical and operational constraints. For instance, to ensure communication and coordination, each follower USV must lie within an effective communication radius *R* of the leader USV. The position decision variable vector for the *N* follower USVs is

(19) $$X\left(r,\theta \right)={\left(r_{1},r_{2},\ldots ,r_{N},\theta_{1},\theta_{2},\ldots ,\theta_{N}\right)}^{T}$$

The following describes three major categories of constraints.

(1) Communication distance constraint

Given the finite size of the USV formation and the limited range of communication modules, each follower USV must remain within distance *R* of the leader USV. Assuming the leader is located at the formation center, this constraint is written as

(20) $$\left\{X\left(r,\theta \right)|0<r_{i}<R\right\},\;i=1,2,\ldots ,N$$

where $r_{i}$ is the radial distance of follower *i* from the leader, and *N* is the number of follower USVs.

(2) Obstacle avoidance constraint

To prevent collisions between follower USVs and known static obstacles, obstacle avoidance constraints are added. Supposing the obstacle is located at $\left(r_{o},\theta_{o}\right)$ in polar coordinates, and the minimum safe distance from the obstacle is $r_{\mathrm{obs}}$, then the constraint is

(21) $$\left\{X\left(r,\theta \right)|r_{i}^{2}+r_{o}^{2}-2r_{i}r_{o}\cos\left(\theta_{i}-\theta_{o}\right)\geq r_{\mathrm{obs}}^{2}\right\},\;i=1,2,\ldots ,N$$

This ensures that the Euclidean distance between follower USV *i* and the obstacle is no less than $r_{\mathrm{obs}}$.

(3) Collision avoidance constraint

Similarly, to prevent mutual interference among follower USVs, the minimum separation distance between any two USVs *i* and *j* must be greater than a predefined margin $r_{\mathrm{safe}}$. This constraint can be handled by pairwise penalty terms or explicitly excluded in the encoding phase of the optimization algorithm. In complex maritime environments, the impact of static obstacles on USV formations cannot be ignored. During the formation optimization process, it is essential to ensure that no follower USV enters the danger zone around an obstacle.

Let the safe buffer radius around the obstacle be defined as $r_{\mathrm{obs}}$, and the obstacle located at $\left(r_{o},\theta_{o}\right)$ in polar coordinates. The avoidance constraint is formulated as

(22) $$\left\{X\left(r,\theta \right)\;|\;r_{i}^{2}+r_{o}^{2}-2r_{i}r_{o}\cos\left(\theta_{i}-\theta_{o}\right)\geq r_{\mathrm{obs}}^{2}\right\},\;i=1,2,\ldots ,N$$

where $\left(r_{o},\theta_{o}\right)$ denotes the position of the obstacle. When the distance between follower USV *i* and the obstacle is less than or equal to $r_{\mathrm{obs}}$, the fitness function is penalized (e.g., set to 0), thereby forcing the optimizer to avoid the region.

## 4. Formation Optimization Strategy Based on Improved Genetic Algorithm

Genetic algorithms (GAs) are intelligent optimization methods inspired by the biological principle of “survival of the fittest”. They possess strong global search capabilities, are widely applicable, and have been effectively used in various path planning and combinatorial optimization problems.

In this work, the genetic algorithm is applied to solve the USV formation optimization problem. The encoding structure, individual fitness evaluation, and selection strategy are tailored for the multi-constraint optimization of USV formation geometry.

### 4.1. Genetic Algorithm Principle and Improvement Strategy

The basic GA workflow includes initial population generation, fitness evaluation, selection, crossover, and mutation. In this work, binary encoding is used, and a floating-point encoding structure is adopted to improve convergence precision. The individual gene vector encodes the decision variables $\left(r_{i},\theta_{i}\right)$ of follower USVs.

The standard steps of GA include the following:

1. Initialization: Generate an initial population randomly within bounds;
2. Fitness evaluation: Calculate the fitness of each individual based on multi-objective function *F*;
3. Selection: Apply roulette or tournament selection based on fitness;
4. Crossover and Mutation: Perform genetic operations to generate offspring individuals;
5. Elitism: Retain the best individuals from previous generations to ensure convergence.

To accelerate convergence, a niche-based replacement strategy is introduced. If the new individual violates constraints or duplicates an elite member, it is penalized or discarded.

### 4.2. Formation Optimization Algorithm Flow

To meet the practical requirements of USV formation deployment, such as high-speed convergence and diversity preservation, this work proposes an improved GA with adaptive mutation strategies and elitism retention.

The entire optimization flow is summarized as follows:

- Step 1: Initialize the population $X_{0}$ and assign random positions to each USV $\left(r_{i},\theta_{i}\right)$.
- Step 2: Evaluate the fitness F(X) for each individual based on Equation (17).
- Step 3: Select parents based on fitness, and apply crossover and mutation to generate new individuals.
- Step 4: Apply abnormality judgment. If $F_{\max}\cdot \alpha <F_{\mathrm{avg}}$, mark as stagnation.
- Step 5: In case of stagnation, apply a large mutation; otherwise, perform a small mutation.
- Step 6: Evaluate and update elite individuals, update generation counter.
- Step 7: If the stopping condition is met, output the optimal solution; else, return to Step 2.

This framework enables adaptive exploration and exploitation, achieving stable convergence to optimal formation configurations that satisfy all detection and collision constraints.

After the selection, crossover, and mutation operations, if the best individual fitness $F_{\max}$ drops significantly, an elitist strategy is applied. Specifically, if

(23) $$F_{\max}\cdot \alpha <F_{\mathrm{avg}}$$

then the current best individual is considered “degenerated” and replaced by the historical best. Here, α is a stagnation threshold factor, typically in the range (0.5,1).

In such a case, the global best solution is used to reinitialize a new individual:

(24) $$X^{\prime }\left(r,\theta \right)=\left\{X_{i}\left(r,\theta \right)\;|\;i=1,2,\ldots ,M\right\}$$

Using the fitness function *F* from Equation (17), the best-performing individual is retained as

(25) $$X_{\mathrm{new}}=\arg\max\left\{F\left(X_{i}\left(r,\theta \right)\right),i=1,2,\ldots ,M\right\}$$

### 4.3. Flowchart of the Formation Optimization Algorithm

**Figure 4:**
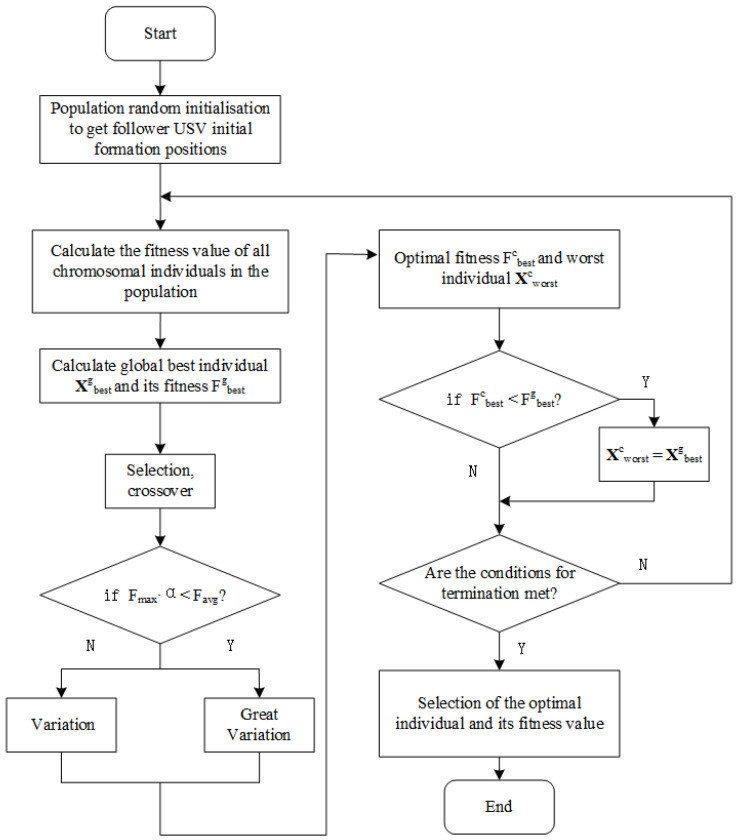
Flowchart of USV formation optimization based on improved GA (N: No, Y: Yes).

The overall process of the improved GA for USV formation design is summarized in Figure 4. The logic includes initialization, fitness evaluation, elite preservation, and convergence judgment.

## 5. Experimental Results

### 5.1. Experimental Setup

To validate the proposed formation optimization strategy, numerical simulations are conducted using MATLAB R2020a. The formation consists of one leader USV and four follower USVs. For comparative purposes, the simulation parameters are aligned with those used in reference [14]. The environment is discretized using a grid step size of 0.1 km.

The maximum detection range of the passive sonar is determined by its quality factor (FOQ), which is fixed at 70 dB. According to Equation (3) this corresponds to a maximum effective detection radius of 3 km:

$$R_{\max}=\frac{1}{\alpha }\cdot \mathrm{FOQ}=\frac{1}{\alpha }\cdot 70\;\left(\mathrm{dB}\right)$$

where α denotes the acoustic attenuation coefficient.

To ensure collision avoidance and formation integrity, as shown in Table 2 the following constraint parameters are defined: formation distance constraint R=2km, inter-vehicle safety distance $r_{\mathrm{safe}}=0.2\;\mathrm{km}$, and detection threshold DT=1dB. The effective detection probability threshold is set to $P_{t}=0.8$. Additionally, obstacles are included in the environment by defining their influence radius $r_{\mathrm{obs}}=0.5\;\mathrm{km}$, simulating environmental interference or topographic shadowing.

### 5.2. Evaluation Metrics

To quantitatively assess the performance of different USV formation patterns in cooperative detection tasks, three evaluation metrics are defined: the detection coverage area $S_{\mathrm{cov}}$, the effective forward detection width $L_{\mathrm{wid}}$, and the overall detection efficiency *F*. These metrics jointly reflect the formation’s spatial distribution, directional detection capacity, and comprehensive effectiveness.

1. Detection coverage area $S_{\mathrm{cov}}$

The detection coverage area represents the total area over which the USV formation maintains an effective probability of detection. It is defined as

(26) $$S_{\mathrm{cov}}=\sum_{(x,y)\in \Omega }A_{\mathrm{cell}}\cdot I\left(P\left(x,y\right)\geq P_{t}\right)$$

where $A_{\mathrm{cell}}$ denotes the area of each discretized grid cell, P(x,y) is the detection probability at point (x,y), $P_{t}$ is the detection threshold, and I(·) is the indicator function which selects only those cells meeting the threshold criterion.

2. Effective forward detection width $L_{\mathrm{wid}}$

The effective forward detection width quantifies the span of the detection field in the direction orthogonal to the formation’s movement. It measures the width along the front line of the formation where the detection probability exceeds the threshold $P_{t}$:

(27) $$L_{\mathrm{wid}}=\max_{y}\left\{x_{2}-x_{1}\;|\;P\left(x,y\right)\geq P_{t}\;\mathrm{for}\;x\in \left[x_{1},x_{2}\right]\right\}$$

This metric highlights the directional detection effectiveness and is crucial in frontal search operations.

3. Overall detection efficiency *F*

To provide a unified assessment, the comprehensive detection efficiency is defined as a weighted sum of normalized area and width metrics:

(28) $$F=\lambda_{1}\cdot \frac{S_{\mathrm{cov}}}{S_{\max}}+\lambda_{2}\cdot \frac{L_{\mathrm{wid}}}{L_{\max}}$$

where $S_{\max}$ and $L_{\max}$ represent the maximum theoretical coverage and width under ideal conditions, and $\lambda_{1}$, $\lambda_{2}$ are weighting coefficients satisfying $\lambda_{1}+\lambda_{2}=1$.

### 5.3. Impact of Weight Parameters on USV Formation Behavior

The weights $u_{1}$ and $u_{2}$ represent the priority levels of performance indicators when a USV formation executes cooperative detection tasks. These weights can be flexibly adjusted according to mission-specific requirements, thereby enabling optimal formation transitions tailored to varying objectives.

To examine the influence of indicator weighting on formation geometry, a comparative simulation study is conducted using three representative weight configurations:

- Pre-optimization (baseline);
- $u_{1}=0.3$, $u_{2}=0.7$;
- $u_{1}=0.7$, $u_{2}=0.3$.

These configurations are selected to illustrate how different preferences between detection coverage area and forward detection width affect the resulting formation pattern.

**Figure 5:**
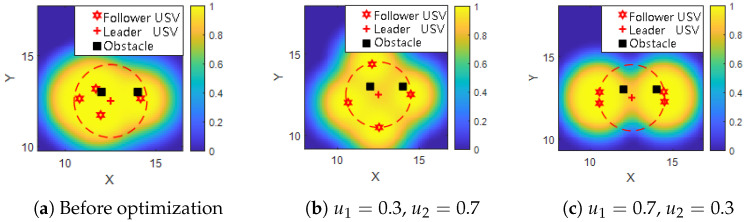
Comparison of USV formation patterns under different optimization conditions. Red stars indicate follower USVs, red crosses indicate the leader USV, and black squares represent obstacles. Color gradients represent the cooperative detection probability distribution, with warmer colors indicating higher probabilities. Dashed ellipses illustrate the effective detection area.

As shown in Figure 5, the “+” symbol represents the leader USV, and “∗” denotes each follower USV. The coordinate system is centered on the leader’s position.

In Figure 5a, prior to optimization, the follower USVs are randomly placed, and some units fall within the influence regions of nearby obstacles, resulting in suboptimal detection performance. After optimization, the USVs are repositioned to avoid obstacle interference, leading to a noticeable improvement in both forward detection width and total effective coverage area.

In Figure 5b, where $u_{2}=0.7$, greater priority is given to maximizing forward detection width. The resulting formation stretches along the direction of movement, and the follower USVs are more widely spaced to expand the sensing front.

Conversely, in Figure 5c, with $u_{1}=0.7$, the optimization favors maximizing the overall detection coverage area. As a result, the formation becomes more compact, maintaining better cohesion among USVs and satisfying constraints on inter-vehicle spacing. This arrangement improves total sensing coverage while enhancing formation coordination.

**Figure 6:**
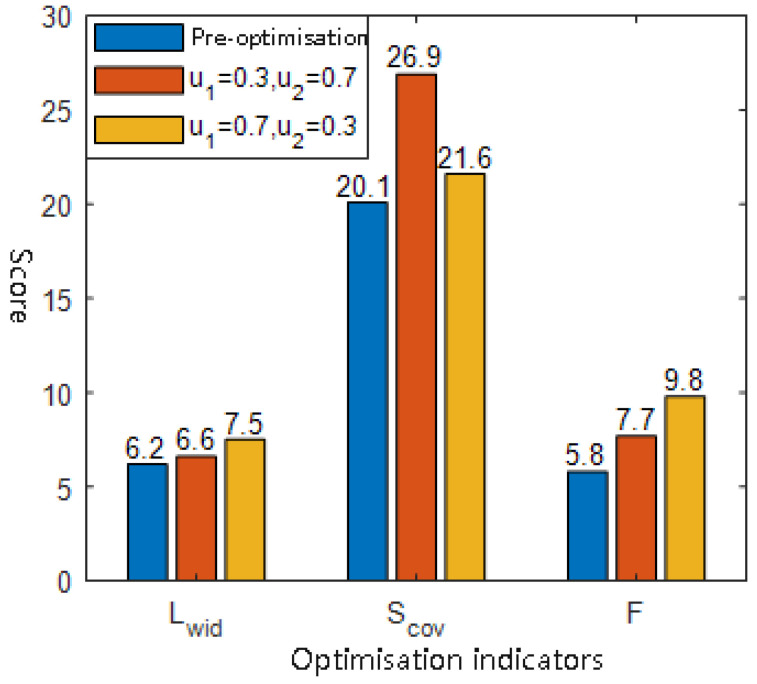
Simulation comparison results of USV formation optimization under different weighting conditions.

The comparative results of the formation optimization simulations are shown in Figure 6. Compared to the unoptimized formation, the proposed method, based on improved bi-level evolutionary optimization, yields a significant increase in the comprehensive detection efficiency *F* of the USV formation.

When $u_{1}=0.7$, the optimization favors maximizing detection coverage area, resulting in a 33.83% increase in $S_{\mathrm{cov}}$, and the overall performance score *F* is improved by 32.76%. In contrast, when $u_{2}=0.7$, which emphasizes forward detection width, the indicator $L_{\mathrm{wid}}$ shows a 20.97% increase relative to the pre-optimized state, and *F* also improves by 68.97%.

These results demonstrate that the proposed formation optimization method can effectively adapt to different task priorities by adjusting the weight coefficients, thereby enhancing the USV formation’s adaptability and sensing effectiveness under complex detection scenarios.

To investigate the influence of static obstacles on USV formation optimization, four obstacles are introduced into the simulation environment. These obstacles are placed near the final positions of the follower USVs after formation optimization.

**Figure 7:**
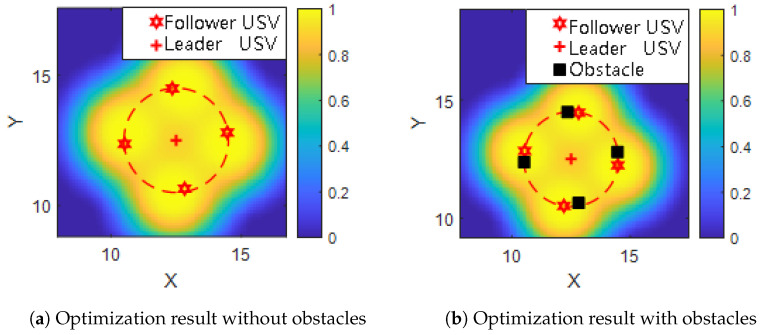
Comparison of USV formation optimization results with and without obstacle interference. Red stars denote follower USVs, red crosses indicate the leader USV, and black squares represent static obstacles, detection probability distribution of a USV formation, the red dotted circle indicates the effective communication range of the leader USV.

As shown in Figure 7, the black squares denote the obstacle locations. In Figure 7a, the formation was optimized in an obstacle-free environment. The USVs are symmetrically distributed around the target point, forming a regular and balanced detection layout.

However, when obstacles are present (Figure 7b), the formation adapts accordingly: some USVs are repositioned to avoid overlapping with obstacle regions, leading to slight deformation of the ideal pattern. Despite the deviation from symmetry, the coverage and spacing constraints are still maintained. This demonstrates the robustness of the proposed optimization strategy in complex environments, where safe navigation and effective detection are simultaneously achieved.

### 5.4. Comparison of Algorithm Performance

To evaluate the performance of different optimization strategies in avoiding local optima and accelerating convergence, a comparative experiment is conducted on five algorithms. All methods are constrained to a maximum of 1000 generations to ensure fairness, and their convergence speed and final fitness values are analyzed.

The algorithms tested include the basic genetic algorithm (GA) [16], basic particle swarm optimization (PSO) [17], Adaptive Particle Swarm Optimization (APSO) [18], Adaptive Genetic Algorithm (AGA) [19], Multi-Objective Task Scheduling Genetic Algorithm (MOTS-GA) [20], and the proposed improved genetic algorithm. All algorithms are configured with a population size of 20. For PSO-based algorithms, the inertia weight is set to ω=0.5, and the learning factors to $c_{1}=c_{2}=2$. For GA-based methods, the crossover rate is set to $p_{c}=0.7$, and the mutation rate to $p_{m}=0.04$. For adaptive versions, the mutation rate $p_{m}$ and crossover rate $p_{c}$ are dynamically adjusted based on their respective adaptive rules. For the improved algorithm, additional parameters are set as p=0.4, crowding factor r=0.6, with weights $u_{1}=0.7$, $u_{2}=0.3$.

The performance of each algorithm is assessed by tracking its convergence curve under the same objective function.

**Figure 8:**
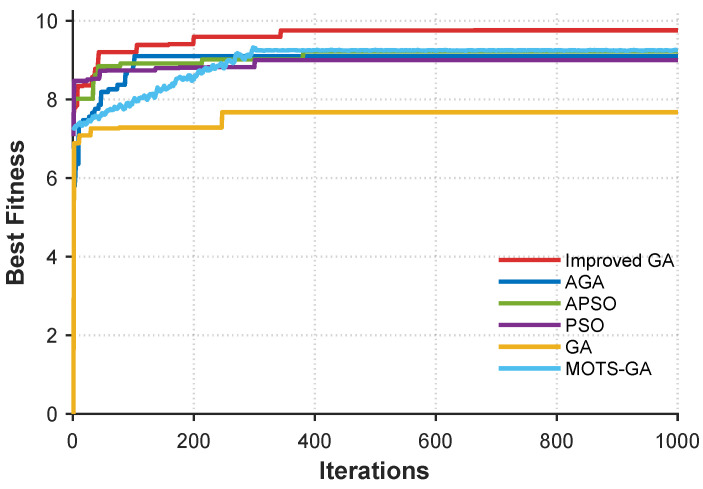
Comparison of convergence curves of five different optimization algorithms: Improved GA, AGA, APSO, PSO, MOTS-GA, and GA. The vertical axis represents the fitness score, and the horizontal axis indicates the number of generations.

As shown in Figure 8, the proposed improved genetic algorithm achieves superior overall detection performance compared to the other four algorithms. Specifically, Table 3 compares five optimization algorithms—PSO, GA, APSO, AGA, and MOTS-GA—based on the effective detection width ($L_{\mathrm{wid}}$), effective detection area ($S_{\mathrm{cov}}$), overall detection efficiency (*F*), convergence time, runtime, and computational complexity. MOTS-GA outperforms all other algorithms in all metrics, with the highest $L_{\mathrm{wid}}$ (7.5 km), $S_{\mathrm{cov}}$ (23.07 km^2^), and *F* (9.85). It also converges the fastest in 84 generations, significantly reducing the time compared to PSO (301 generations). The runtime for MOTS-GA is approximately 15.2 s, slightly slower than the improved GA (12.5 s). All algorithms share a computational complexity of O(n·v·m), where n=20, v=8, and m=1000, reflecting the dependence on population size, variable count, and maximum generations. MOTS-GA demonstrates superior detection performance, efficiency, and faster convergence, making it the most effective solution for the USV formation optimization task.

From a convergence perspective, the improved GA also demonstrates faster convergence speed. While basic GA-based methods may suffer from premature convergence, the adaptive adjustment strategy adopted in the improved GA effectively enhances the global search capability by adjusting crossover and mutation parameters. Despite the higher convergence speed of APSO, its detection performance is relatively inferior.

These results confirm that the proposed improved GA offers better global search performance and convergence reliability in the context of USV formation optimization for cooperative detection missions.

### 5.5. Performance Comparison of Different Fleet Sizes

To investigate the performance of the USV fleet in different scales, we design simulations with three different fleet sizes:

- Small-Scale Fleet: 1 leader USV and 3 follower USVs (total of 4 USVs).
- Medium-Scale Fleet: 1 leader USV and 6 follower USVs (total of 7 USVs).
- Large-Scale Fleet: 1 leader USV and 10 follower USVs (total of 11 USVs).

**Figure 9:**
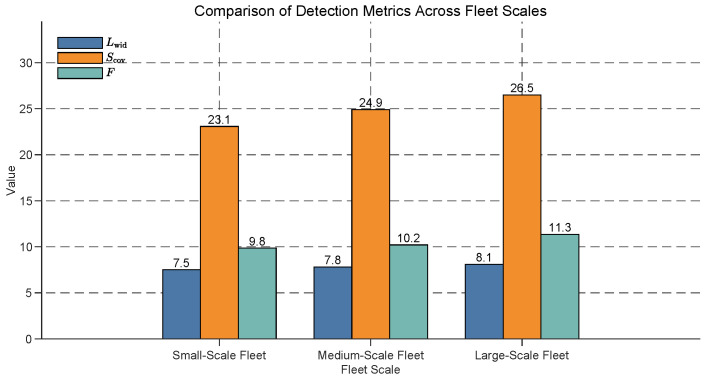
Comparison of detection metrics across different fleet scales. The effective detection width ($L_{\mathrm{wid}}$), effective detection area ($S_{\mathrm{cov}}$), and overall detection efficiency (*F*) are shown for small-scale, medium-scale, and large-scale fleets. All values increase with the fleet scale, with *F* showing the largest relative improvement (15.2% from small scale to large scale).

Figure 9 illustrates the comparison of detection metrics across small-scale, medium-scale, and large-scale fleets. The effective detection width ($L_{\mathrm{wid}}$), effective detection area ($S_{\mathrm{cov}}$), and overall detection efficiency (*F*) all exhibit a clear upward trend with increasing fleet scale. Specifically, $L_{\mathrm{wid}}$ rises from 7.5 in the small-scale fleet to 8.1 in the large-scale fleet (an 8% increase), $S_{\mathrm{cov}}$ increases from 23.07 to 26.5 (a 14.8% increase), and *F* improves from 9.85 to 11.35 (a 15.2% increase). These results suggest that larger fleets enhance detection performance, with the overall detection efficiency (*F*) showing the most significant relative improvement, likely due to the greater resource availability and improved coordination in larger fleets.

### 5.6. Impact of Different Obstacle Densities on Formation Optimization

In practical applications, the density of obstacles may affect the formation optimization of the USV fleet. Therefore, we design simulations with the following obstacle densities:

- Low Obstacle Density: A small number of obstacles in each region.
- Medium Obstacle Density: A moderate number of obstacles in each region.
- High Obstacle Density: A large number of obstacles in each region.

**Figure 10:**
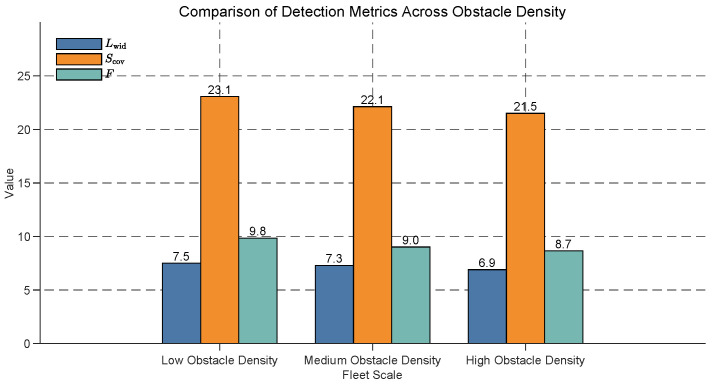
Comparison of detection metrics across different obstacle densities for USV fleet formation optimization. The effective detection width ($L_{\mathrm{wid}}$), effective detection area ($S_{\mathrm{cov}}$), and overall detection efficiency (*F*) are shown under low, medium, and high obstacle densities. All metrics decrease as obstacle density increases, with $S_{\mathrm{cov}}$ showing the largest relative reduction (6.9% from low to high density).

The impact of obstacle density on USV fleet formation optimization is examined, with results shown in Figure 10. The effective detection width ($L_{\mathrm{wid}}$), effective detection area ($S_{\mathrm{cov}}$), and overall detection efficiency (*F*) are compared across low, medium, and high obstacle densities. As obstacle density increases, all metrics exhibit a consistent decline. Specifically, $L_{\mathrm{wid}}$ decreases from 7.5 at low density to 6.9 at high density (an 8.0% reduction), $S_{\mathrm{cov}}$ drops from 23.1 to 21.5 (an 6.9% reduction), and *F* falls from 9.8 to 8.7 (an 11.2% reduction). These results indicate that higher obstacle densities significantly impair the detection performance of USV fleets, with $S_{\mathrm{cov}}$ experiencing the largest relative decline, likely due to the increased occlusion and restricted coverage in denser environments. This underscores the need for robust obstacle avoidance and detection strategies in high-density scenarios, such as ports or busy waterways.

### 5.7. Adaptability of Formation Optimization Algorithm to Speed Variations

To investigate the impact of speed on formation optimization, we design three different speed conditions:

- Low-Speed Sailing: USVs perform the task at a lower speed.
- Medium-Speed Sailing: USVs perform the task at a moderate speed.
- High-Speed Sailing: USVs perform the task at a higher speed.

**Figure 11:**
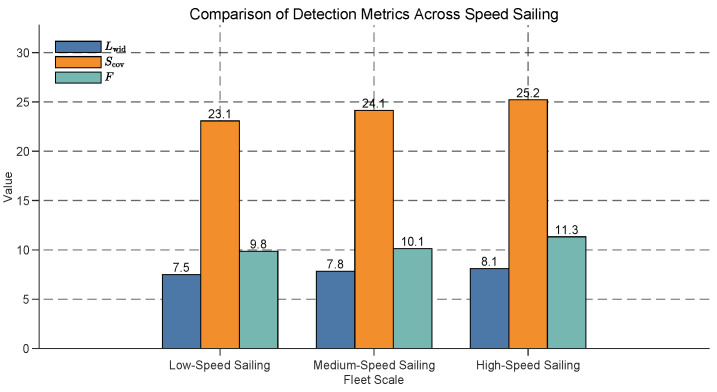
Comparison of detection metrics across different sailing speeds for USV fleets. The effective detection width ($L_{\mathrm{wid}}$), effective detection area ($S_{\mathrm{cov}}$), and overall detection efficiency ($F_{\mathrm{eff}}$) are shown for low-speed, medium-speed, and high-speed sailing conditions. All metrics increase with sailing speed, with $F_{\mathrm{eff}}$ showing the largest relative improvement (14.9% from low speed to high speed).

The influence of sailing speed on USV fleet detection performance is analyzed, with results presented in Figure 11. The effective detection width ($L_{\mathrm{wid}}$), effective detection area ($S_{\mathrm{cov}}$), and overall detection efficiency ($F_{\mathrm{eff}}$) are evaluated under low-speed, medium-speed, and high-speed sailing conditions. All metrics exhibit a consistent upward trend with increasing sailing speed. Specifically, $L_{\mathrm{wid}}$ rises from 7.5 at low speed to 8.1 at high speed (a 8.0% increase), $S_{\mathrm{cov}}$ increases from 23.1 to 25.2 (a 9.1% increase), and $F_{\mathrm{eff}}$ improves from 9.8 to 11.3 (a 14.9% increase). These findings demonstrate that higher sailing speeds significantly enhance the detection capabilities of USV fleets, with $F_{\mathrm{eff}}$ showing the most substantial relative improvement, likely due to enhanced maneuverability and broader coverage at higher speeds. This suggests that optimizing sailing speed can be a critical strategy for improving detection efficiency in time-sensitive missions, such as search-and-rescue operations, although considerations of energy consumption and stability must also be addressed.

### 5.8. Impact of Formation Constraints on Optimization

In practical applications, formation constraints such as minimum distance between USVs, safe collision distance, and formation distance play a crucial role in the optimization process. To study the impact of these constraints, we design simulations with the following conditions:

- Min Distance Between USVs ($R_{\min}$): The minimum allowed distance between two USVs in the formation.
- Safe Collision Distance ($r_{\mathrm{safe}}$): The safety distance to avoid collision between USVs.
- Formation Distance (R): The overall distance between the leader and follower USVs in the formation.

**Figure 12:**
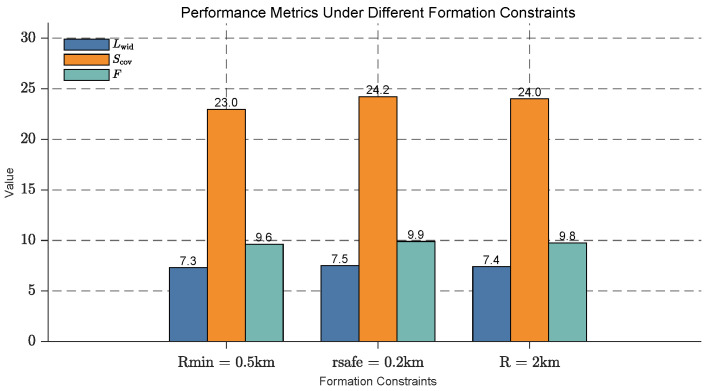
Performance metrics of USV fleet under different formation constraints, including effective detection width ($L_{\mathrm{wid}}$), effective detection area ($S_{\mathrm{cov}}$), and overall detection efficiency (*F*) for three different conditions: $R_{\min}=0.5\;\mathrm{km}$, $r_{\mathrm{safe}}=0.2\;\mathrm{km}$, and R=2km. The bars represent the values of these metrics for each formation constraint.

The influence of formation constraints on the USV fleet’s detection performance is analyzed, with results presented in Figure 12. The effective detection width ($L_{\mathrm{wid}}$), effective detection area ($S_{\mathrm{cov}}$), and overall detection efficiency (*F*) are evaluated under three different formation constraints: $R_{\min}=0.5\;\mathrm{km}$, $r_{\mathrm{safe}}=0.2\;\mathrm{km}$, and R=2km.

All metrics exhibit an upward trend as the formation constraints are relaxed. Specifically, $L_{\mathrm{wid}}$ rises from 7.3 to 7.5 (a 2.7% increase), $S_{\mathrm{cov}}$ increases from 23.0 to 24.2 (a 5.2% increase), and *F* improves from 9.6 to 9.9 (a 3.1% increase). These findings demonstrate that relaxing formation constraints significantly enhances the detection capabilities of the USV fleet, with $S_{\mathrm{cov}}$ showing the most substantial relative improvement, likely due to a more dispersed formation and wider coverage.

### 5.9. The Parameter Sensitivity Analysis

The parameter sensitivity analysis presented in Table 4 identifies key factors influencing the model’s performance. The population size (*n*) has a moderate impact, with an optimal value of 20. Crossover probability ($p_{c}$) and mutation probability ($p_{m}$) both exhibit high sensitivity, with optimal values of 0.7 and 0.04, respectively, suggesting that these parameters significantly affect the model’s efficiency and convergence. The large mutation probability ($p_{L}$) shows a critical impact, highlighting its importance in exploring the solution space, with an optimal value of 0.4. The density factor (α) has a moderate impact, suggesting that adjusting it can improve the solution quality. Lastly, the max iterations parameter shows low sensitivity, indicating that performance is less affected by the iteration limit within the given range.

### 5.10. Robustness Analysis of Improved GA in Varying Environments

The performance of the improved GA algorithm under dynamic conditions shows a notable decline in fitness and coverage when external factors such as dynamic obstacles and communication delays are introduced. As shown in Table 5 in baseline conditions, the algorithm achieves a fitness score of 9.85 and a coverage of 23.07, both indicating optimal performance. However, when dynamic obstacles are present, fitness decreases to 9.42, coverage drops to 22.05, and the success rate falls to 96%, reflecting a degradation of 4.2%. Communication delays (200 ms) further reduce fitness to 9.15 and coverage to 21.35, with an 88% success rate and a degradation of 6.5%. The presence of environmental factors like currents results in a slight improvement in performance (fitness = 9.53, coverage = 22.65), demonstrating the algorithm’s resilience. When combined factors are considered, the overall degradation becomes more pronounced (fitness = 8.87, coverage = 20.95), showing a 10.5% reduction in the success rate.

## 6. Conclusions

This study addresses the formation planning problem of USV formations in maritime detection missions and proposes an improved genetic algorithm-based formation optimization strategy. First, a cooperative detection probability model for USV formations was constructed based on discretized acoustic target detection modeling, thereby establishing a probabilistic sensing framework. A multi-indicator optimization objective was then defined, incorporating forward detection width and effective coverage area to enhance overall detection capability. A weighted multi-objective fitness function was introduced to reflect varying mission priorities, enabling adaptive formation adjustment. To ensure obstacle avoidance and navigational safety, static obstacles were incorporated into the environmental model. A grid-based constraint mechanism was designed to prevent follower USVs from entering obstacle zones, and genetic operators were improved with adaptive crossover and mutation strategies to enhance global search performance. Simulation results demonstrated that the improved genetic algorithm significantly outperforms conventional methods in terms of detection performance and convergence efficiency. Compared to unoptimized formations, the proposed method achieved noticeable improvements across multiple indicators. When the coverage area was prioritized ($u_{1}=0.7$), the effective detection area increased by 32.76%. When forward detection width was prioritized ($u_{2}=0.7$), the formation’s front width increased by 20.97%. These results verify the effectiveness and robustness of the proposed method in enhancing the sensing performance of USV formations under complex mission conditions.

## Figures and Tables

**Table 1:** Summary of Acoustic Model Parameters.

Parameter	Description
SL	Source Level
TL	Transmission Loss
NL	Noise Level
DI	Directivity Index
FOQ	Sound Quality Factor
$\sigma^{2}$	Noise Variance
$P_{f}$	False Alarm Probability

**Table 2:** Key parameters used in the USV formation optimization simulation.

Parameter	Symbol	Value
Grid resolution	Δx, Δy	0.1 km
Sonar quality factor	FOQ	70 dB
Maximum detection range	$R_{\max}$	3 km
Formation distance constraint	*R*	2 km
Detection threshold	DT	1 dB
Effective detection probability threshold	$P_{t}$	0.8
Safety distance between USVs	$r_{\mathrm{safe}}$	0.2 km
Obstacle influence radius	$r_{\mathrm{obs}}$	0.5 km

**Table 3:** Comparison of experimental results for five optimization algorithms with time and computational complexity.

Indicator	PSO	GA	APSO	AGA	MOTS-GA	Improved GA
$L_{\mathrm{wid}}$	7.2	7.2	7.2	7.3	7.4	7.5
$S_{\mathrm{cov}}$	22.77	21.74	21.48	21.93	22.53	23.07
*F*	9.04	8.92	8.91	9.15	9.35	9.85
Converge	301	246	244	102	253	84
Runtime (s)	18.7	16.5	16.2	12.9	15.2	12.5
Complexity (*O*)	O(n·v·m)	O(n·v·m)	O(n·v·m)	O(n·v·m)	O(n·v·m)	O(n·v·m)

**Table 4:** Parameter sensitivity analysis.

Parameter	Range	Optimal	Impact
Population size (*n*)	10–50	20	Moderate
Crossover prob ($p_{c}$)	0.5–0.9	0.7	High
Mutation prob ($p_{m}$)	0.01–0.1	0.04	High
Large mutation prob ($p_{L}$)	0.3–0.6	0.4	Critical
Density factor (α)	0.3–0.8	0.5	Moderate
Max iterations	500–2000	1000	Low

**Table 5:** Performance under dynamic conditions for the improved GA.

Condition	Fitness	Coverage	Success Rate	Degradation
Baseline	9.85	23.07	100%	0%
Dynamic obstacles	9.42	22.05	96%	4.2%
Comm delay (200 ms)	9.15	21.35	93%	6.5%
Currents (0.2 m/s)	9.53	22.65	98%	3.2%
Combined factors	8.87	20.95	89%	10.5%

## Data Availability

The datasets presented in this article are not readily available due to technical limitations and ongoing data processing. Requests to access the datasets should be directed to the corresponding author.
